# How Mouse Macrophages Sense What Is Going On

**DOI:** 10.3389/fimmu.2016.00204

**Published:** 2016-06-02

**Authors:** Klaus Ley, Akula Bala Pramod, Michael Croft, Kodi S. Ravichandran, Jenny P. Ting

**Affiliations:** ^1^Division of Inflammation Biology, La Jolla Institute for Allergy and Immunology, La Jolla, CA, USA; ^2^Department of Bioengineering, University of California San Diego, La Jolla, CA, USA; ^3^Division of Immune Regulation, La Jolla Institute for Allergy and Immunology, La Jolla, CA, USA; ^4^Department of Microbiology, Immunology, and Cancer Biology, University of Virginia, Charlottesville, VA, USA; ^5^Department of Genetics, The Lineberger Comprehensive Cancer Center, University of North Carolina at Chapel Hill, Chapel Hill, NC, USA

**Keywords:** macrophages, pathogens, immunity, defense, inflammation

## Abstract

Macrophages are central to both innate and adaptive immunity. With few exceptions, macrophages are the first cells that sense trouble and respond to disturbances in almost all tissues and organs. They sense their environment, inhibit or kill pathogens, take up apoptotic and necrotic cells, heal tissue damage, and present antigens to T cells. Although the origins (yolk sac versus monocyte-derived) and phenotypes (functions, gene expression profiles, surface markers) of macrophages vary between tissues, they have many receptors in common that are specific to one or a few molecular species. Here, we review the expression and function of almost 200 key macrophage receptors that help the macrophages sense what is going on, including pathogen-derived molecules, the state of the surrounding tissue cells, apoptotic and necrotic cell death, antibodies and immune complexes, altered self molecules, extracellular matrix components, and cytokines, including chemokines.

## Introduction

Macrophages are central to proper functioning of the immune system (1–4). Resting tissue macrophages make ornithine (by arginase) and promote repair, called the M2 phenotype (1, 5). Tissue macrophages arise from embryonic precursors and from blood monocytes at varying proportions (6). At the extremes are microglia cells, which are entirely embryonic-derived (7) and intestinal macrophages, which are entirely monocyte-derived (8) with other organs being populated by mixtures of both. When these tissue macrophages sense trouble (1), they express *IL12* and *iNOS*, and become M1 macrophages (5). Macrophages express hundreds of sensor molecules to identify their surroundings (cells, extracellular matrix, and tissue), the state of the tissue (normal and healthy, apoptotic, ischemic, necrotic, altered, or otherwise distressed), metabolites [oxygen, pH, lactic acid, glucose, free fatty acids, sphingosine-1-phosphate (*S1P*)], lipoproteins (LDL, HDL, and their derivatives), antibodies (IgG, immune complexes), complement (*C3a, C3bi, C5a*), cytokines (*IFN-*γ*, IL4, IL17*), and pathogens (bacteria, viruses, fungi, parasites, and their products). Dendritic cells, another key sensor of pathogens, act in concert with the macrophages in initiating an adaptive immune response. Dendritic cells activate the adaptive immune system by antigen presentation to naive T cells in the context of co-activating receptors. These T cells become licensed to traffic to the infected or inflamed tissue (9, 10), where they see antigen presented by macrophages (11) and differentiate (CD4: Th1, Th2, Th17, Treg, TFH and others, CD8: CTL and others). Many products of the adaptive immune system, such as cytokines, can further activate macrophages and alter their function; for example, *IFN-*γ enhances M1 polarization (12) and *IL4* induces alternative activation (13), thus enhancing M2 polarization (1, 14).

This review is focused on the inputs to the macrophage and dendritic cell system: the cell surface and intracellular receptors by which these cells sense what is going on. For details of the signaling networks and effector systems downstream of these receptors, like TRAFs (15–17), *NFkB* (18, 19), or inflammasome assembly (20), the reader is referred to other reviews. Although there are human homologs for almost all receptors discussed here, this review is entirely based on mouse data. The innate immune system and macrophages in particular are under enormous evolutionary pressure shaped by the environment and infectious organisms that differ between mice and humans.

## Tissue Input at Steady State

At steady state, signals from host tissue cells result in tissue-specific gene expression profiles (21). Langerhans cells of the skin, alveolar macrophages, Kupffer cells of the liver, microglia cells of the CNS, osteoclasts, dendritic cells of the thymus, and other lymphoid organs all have specialized functions and phenotypes. This suggests that tissue-derived signals control the development and polarization of tissue-specific macrophage phenotypes. The first tissue cues were identified in osteoclasts (22) and peritoneal macrophages (23, 24). A key inducer of the peritoneal macrophage phenotype is retinoic acid produced by intestinal cells, which is recognized by the nuclear receptor retinoic acid receptor-β (*RAR-*β). Retinoic acid induces the transcription factor *GATA6*, which in turn controls a number of effector molecules (23, 24). Interestingly, *RAR-*β ligation also induces arginase-1, the defining enzyme of M2 macrophages, underscoring that resting peritoneal macrophages, like macrophages in other tissues, are in the default “healing” M2 state. A second known M2-polarizing stimulus is lactic acid, produced by hypoxic cells in cancer (25). This is consistent with the known M2 phenotype of myeloid-derived suppressor cells (26), a macrophage type found in cancers. Under homeostatic conditions, M2 polarization is maintained by *TGF-*β, and *TGF-*β receptor-1 seems to be the major factor in determining microglia phenotype (27). Under inflammatory conditions, *TGF-*β induces a state of deactivation that is different from M2, and promotes the resolution of inflammation. *TGF-*β*2* is produced by resident peritoneal macrophages (28) and might play a significant role in homeostatic maintenance of surrounding tissues.

**Figure 1:**
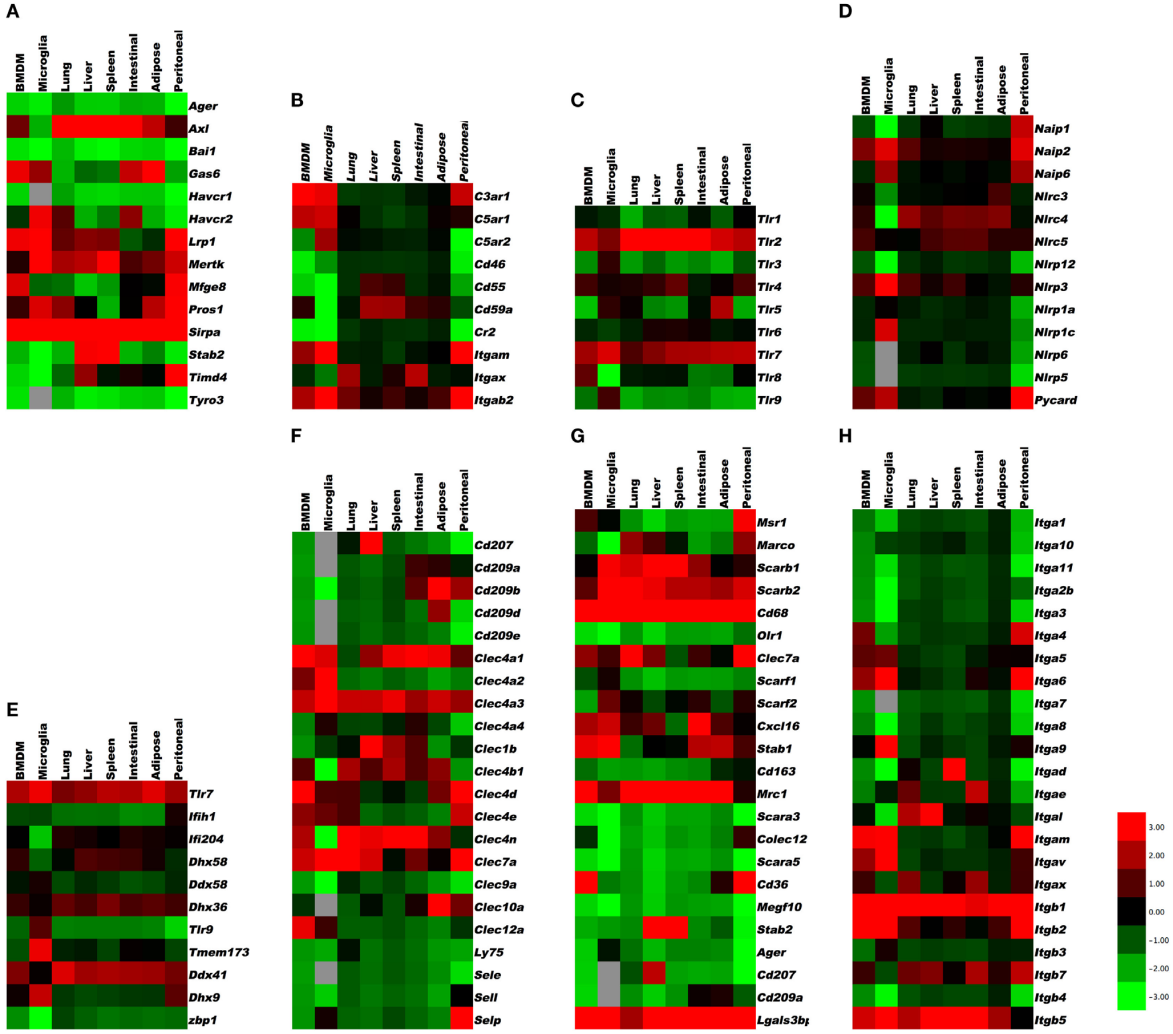

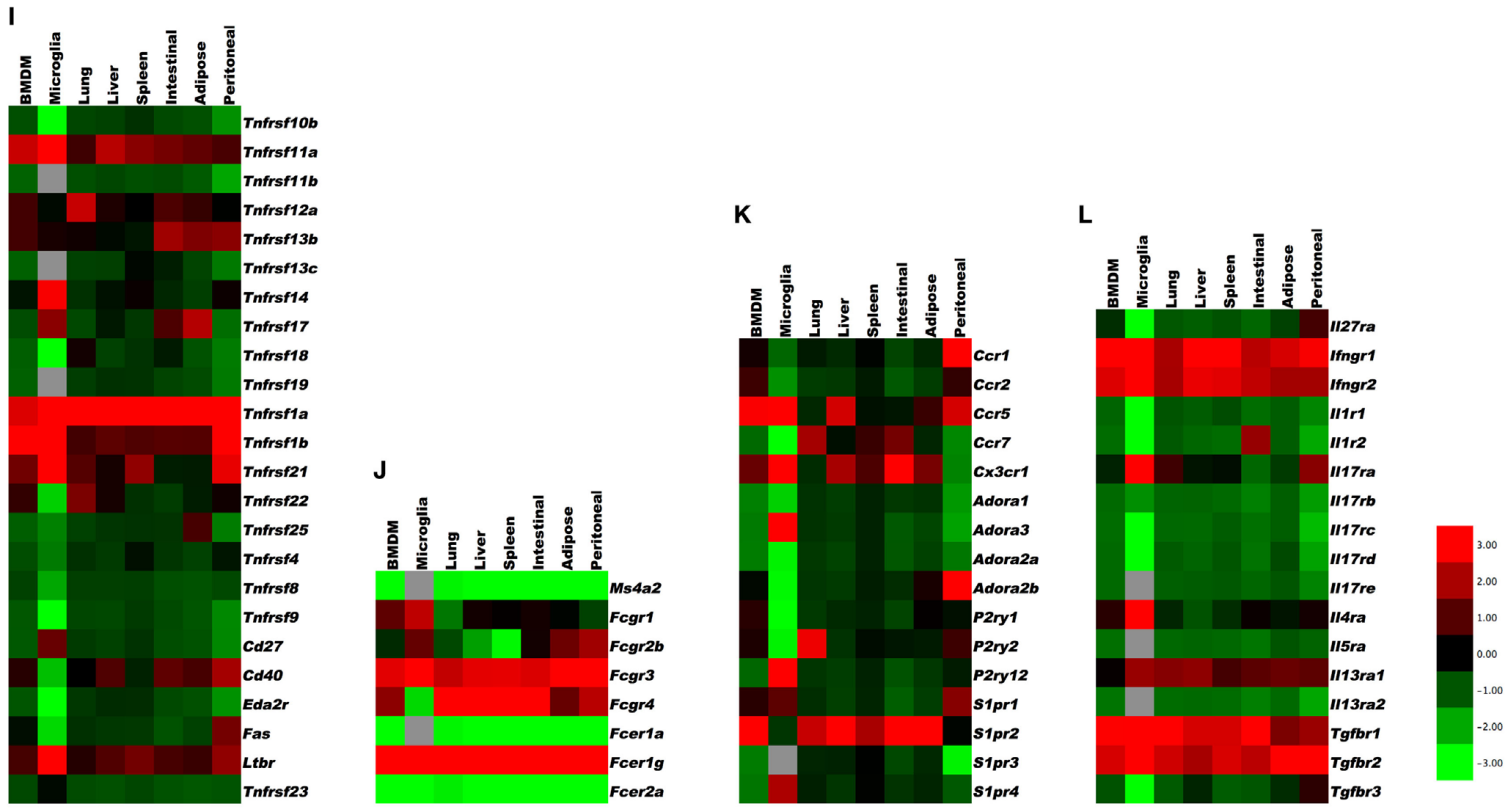
**Gene expression in resident tissue-specific macrophages (microglia, lung, liver, spleen, intestinal, adipose) and bone marrow-derived macrophages (BMDM) for comparison**. Heat maps based on hierarchical clustering results from microarray and RNA-Seq data for **(A)** apoptosis receptors, **(B)** complement receptors, **(C)** toll-like receptors, **(D)** NOD-like receptors, **(E)** RNA and DNA sensors, **(F)** C-type lectins, **(G)** scavenger receptors, **(H)** integrins, **(I)** tumor necrosis factor receptor superfamily, **(J)** Fc receptors, **(K)** G-protein-coupled receptors, and **(L)** cytokine receptors. Gene expression levels are expressed in log_2_ values, differences shown in color scale after *Z*-score transformation. Colors scale bar ranges from red (+3) to green (−3). Light gray indicates undetectable.

To provide a first glimpse at the expression of almost 200 “input” receptors, we compiled heat maps from published data sets on mouse peritoneal macrophages (large, small, and thioglycollate-elicited), microglia (27) (data set GSE62826), and the macrophages from lung, liver, spleen, intestinal, adipose tissue, and bone marrow (23) (data sets GSE56682, 56683, 56684) and (29) (data set GSE47049). The transcriptome data sets were accessed through the Gene Expression Omnibus site^1^ to examine the gene expression profiles in tissue-specific macrophages for 12 categories of receptors [apoptotic cell receptors, complement receptors, toll-like receptors (*TLR*), NOD-like receptors (*NLR*), RNA and DNA receptors, C-type lectins, scavenger receptors, selected cytokine receptors, TNF receptor superfamily members, Fc receptors, selected G-protein-coupled receptors, and integrins]. Cross-platform comparison of gene expression profiles between microarray (GSE47049, GSE56682, GSE56683, and GSE56684) and RNA-Seq (GSE62826) was done on normalized data provided by the authors on the GEO site. The normalized values from different platforms were scaled by log_2_ transformation for comparison through generation of heat maps (30–32). In brief, MAS5.0 (Affymetrix) and quantile normalized (Illumina GUI) probe data available for the microarray platforms, i.e., GSE47049 and GSE56682/83/84 from Affymetrix Mouse Genome 430 2.0 Array and Illumina mouse expression bead chip, respectively, were annotated by R/Bioconductor packages^2^ and annotation files downloaded from the GEO site (GPL6105 and GPL6887). The normalized RPKM gene expression levels from the Illumina RNA-sequencing (Illumina HiSeq 2000) dataset (GSE62826) were obtained from the GEO site. Whenever a gene was represented by more than one probe set or entry, the average of the pooled expression values was used. Furthermore, the average value of the genes across the samples for a tissue from different datasets was calculated for log_2_ transformation. Cluster 3.0 with pair-wise complete linkage was used to perform the hierarchical analysis of log_2_ transformed expression values of around 200 genes using mean-centered gene expression vectors and visualized with the Java TreeView 10.6 (32, 33). The graphical representation of output as heat maps is presented in color scale from green to red (*Z*-score transformation), where red and green indicate higher and lower expression levels, respectively (Figure 1). Non-expressed genes are shown in light gray.

## Receptors for Apoptotic Cells

In many tissues, there is continuous turnover of cells, where the majority of them die by the process of caspase-dependent apoptosis. When cells undergo apoptosis, their corpses are phagocytosed by macrophages in a process referred to as efferocytosis (34–36). In addition to macrophages, epithelial cells and fibroblasts clear apoptotic cells. Apoptotic cells expose phosphatidylserine (PtdSer) on the outer leaflet of their plasma membrane, which serves as a recognition signal (Eat-me). Apoptotic cells, at the earliest stages of death, also release ATP and other molecules that serve as diffusible “find-me” signals to attract macrophages to their proximity. The find-me signals are sensed by tissue-resident macrophages via some G-protein-coupled receptors, such as *P2Y2* sensing ATP and UTP released from apoptotic cells, and *CX3CR1* sensing *CX3CL1* released from apoptotic cells.

There are several recognition systems for PtdSer (Table 1). Routine, homeostatic uptake of apoptotic cells is inherently anti-inflammatory and helps keep the local tissue inflammation to a very minimal (or below detection) level even in tissues where there is a very high cell turnover (such as the bone marrow or thymus). This is in part achieved through the release of mediators, such as *TGF-*β*, IL10*, and prostaglandin E2 (*PGE2*) from macrophages that engage apoptotic cells. *TGF-*β and *IL10* have anti-inflammatory functions. Depending on the receptor triggered (*EP1-EP4*), *PGE2* can have pro- and anti-inflammatory functions, modulate pain sensation, and can activate mast cells (37). See Ref. (38, 39) for more details on apoptotic cell clearance. α*V*β*3* integrin (see Integrins) recognizes PtdSer through *MFGE8*. *RAGE* and stabilin 2 also bind PtdSer and are listed under scavenger receptors (below). *Tyro3, Axl*, and *Mer* bind PtdSer through *GAS6* (gene name *Gas6*) or protein S (gene name *Pros1*). *Mertk*, the gene encoding *Mer* tyrosine kinase, distinguishes macrophages from dendritic cells and has been proposed as a universal mouse macrophage marker (40) when used in combination with other markers like F4/80, *CD68*, or *CD11b*. *CD91*, also known as LDL receptor-related protein *LRP*, is a receptor for calreticulin, which is exposed in cells undergoing ER stress and apoptosis. *SIRP*α (gene name *Sirpa*) can detect *CD47* on apoptotic lymphocytes. The results presented in Figure 1A summarize the expression of genes related to apoptotic cell recognition and uptake. The clearance of apoptotic cells by phagocytes is counterbalanced by mechanisms that limit detrimental effects, such as production of reactive oxygen species. Also, efferocytic receptors, such as *Mer* and *CD11b*, can be downregulated, a process that limits further signaling and uptake of apoptotic cells.

**Table 1:** **PtdSer recognition receptors**.

Gene name	PtdSer receptors	Function	PtdSer binding
*Bai1*	BAI1	Upstream of ELMO1-DOCK180-RAC, engulfment	Direct PtdSer binding
*Havcr1*	TIM1	Also hepatitis A virus receptor	Direct PtdSer binding by metal ion-dependent binding site
*Havcr2*	TIM3	Also for antigen cross-presentation	
*Timd4*	TIM4	Only tethering of apoptotic cells without direct signaling to engulfment	
*Tyro3*	Tyro3	Tyrosine kinase receptor	Indirect PtdSer binding through Gas6 or protein S
*Axl*	Axl	Tyrosine kinase receptor	
*Mertk*	Mer	Tyrosine kinase receptor, universal mouse macrophage marker	

## Receptors for Necrotic Cells and Modified Self

Macrophages sense when tissue cells are distressed by events, such as ischemia (pro-angiogenic, pro-inflammatory), reperfusion (pro-inflammatory), tissue necrosis (pro-inflammatory), and altered self induced by enzymatic modification resulting in (lipo) protein citrullination (41), oxidation (42), or nitrosylation (43). Many of the receptors for altered self are among the category of cell surface molecules that are generally referred to as scavenger receptors (see below). Macrophages express many scavenger receptors that are capable of recognizing these necrotic or altered self-molecule on stressed cells within tissues.

Necrotic cells are opsonized by complement factors that are recognized through complement receptors (44, 45) (Table 2; expression in Figure 1B). The scavenger receptor *RAGE* (see below) also recognizes apoptotic cells and triggers pro-inflammatory mechanisms. The *IL33* receptor ST2 detects *IL33* released by necrotic cells. *CD24* on macrophages binds *HMGB1*, which in turn binds necrotic cells. Mincle (*Clec4e*) and *CLEC9A* are two C-type lectins (listed below) involved in the uptake of necrotic cells. *TLRs* (see below) can recognize the nuclear protein *HMGB1*, the Sin3A-Associated Protein *SAP130*, the heat shock protein *HSP90*, DNA, and urate crystals, all of which can be exposed on or released from necrotic cells.

**Table 2:** **Complement receptors**.

Gene name	Complement receptors	CD name	Function
*Cr1*	CR1	CD35	Binds C3b and C4b, processes opsonized particles
*Cr2*	CR2	CD21	Binds C3bi, C3d. In the mouse, both CR1 and CR2 are derived from the *Cr2* locus by alternative splicing (44)
*Itgam* *Itgb2*	CR3	CD11b/CD18	Obligatory heterodimer, binds C3bi, denatured proteins (and many other ligands)
*Itgax* *Itgb2*	CR4	CD11c/CD18	Obligatory heterodimer, binds C3bi, denatured proteins
*C3ar1*	C3aR		Binds C3a
*C5ar1*	C5aR1	CD88	Binds C5a, activates macrophages
*C5ar2*	C5aR2		Binds C3a, C5a, and their desarginated inactive forms
*Cd59a*	CD59	CD59	Inhibits the membrane attack complex
*Cd46*	MCP	CD46	CD46 and CD55 inactivate the C3/C5 cleaving enzymes
*Cd55*	Decay-accelerating factor DAF	CD55	

## Pathogen Receptors

Pathogen sensors on macrophages can detect bacterial, viral, fungal, and parasite infections. These receptors bind pathogens directly or recognize their products. They form six main classes: *TLR* (17, 46), *NLR* (20, 47), receptors for intracellular RNA, including *RIG-I* like receptors (*RLR*) (48), receptors for intracellular DNA, including *STING* (49, 50), C-type lectins (51, 52), and scavenger receptors (53).

## Toll-Like Receptors

Toll-like receptors (Table 3) are a family of homodimeric transmembrane receptors that recognize bacterial, viral, and fungal products. Some also have endogenous ligands. *TLRs 1, 2, 4, 5*, and *6* are expressed on the plasma membrane, whereas *TLRs 3, 7, 8*, and *9* are expressed in endosomes (17, 54). Most *TLRs* signal through the *MyD88* pathway, ultimately resulting in *NFkB* activation, except *TLR3*, which signals through the intermediary *TRIF* resulting in type I interferon production. *TLR4* signals through both *MyD88* and *TRIF*. (Note: *TLR10* is not expressed in mice).

**Table 3:** **Toll-like receptors**.

Gene name	TLR	Ligands	Location and function	Dimerization
*Tlr1*	TLR1	Bacterial lipoproteins	Cell surface. Activates NFkB through TIRAP, MyD88, IRAKs, TRAF6	Heterodimerizes with TLR2
*Tlr2*	TLR2	Lipoproteins, LTA, PGN, lipoarabinomannan, viral proteins, fungal mannans, trypanosome tGPI–mutin	Cell surface. Activates NFkB through TIRAP, MyD88, IRAKs, and TRAF6	Heterodimerizes with TLR1 or 6 or CD36, homodimer in endosomes
*Tlr3*	TLR3	Viral RNA	Intracellular vesicles, activates NFkB through TRIF-TAK1 and type I interferons through TRIF-TRAF3	Homodimer in endosomes
*Tlr4*	TLR4	LPS, fungal mannans, glycoinositol-phospholipids of parasites, oxLDL	Cell surface and phagosomes, activates NFkB through TIRAP, MyD88, IRAKs, and TRAF6, and induces type 1 interferons through TRAM-TRIF-IRF3	Homodimer
*Tlr5*	TLR5	Flagellin	Cell surface. Activates NFkB through MyD88, IRAKs, TRAF6	Homodimer
*Tlr6*	TLR6	TLR2/6: bacterial lipoproteins, fungal zymosan, β-glucan	Cell surface. Activates NFkB through TIRAP, MyD88, IRAKs, TRAF6	Heterodimerizes with TLR2
*Tlr7*	TLR7	Bacterial, viral, and fungal RNA	Intracellular vesicles, activates NFkB through MyD88, TRAF6, induces type I interferons via MyD88	Homodimer in endosomes
*Tlr8*	TLR8	Viral RNA	Intracellular vesicles	
*Tlr9*	TLR9	Bacterial, viral, fungal, and parasite DNA	Intracellular vesicles, activates NFkB through MyD88, TRAF6, induces type I interferons via MyD88	Homodimer in endosomes

The intracellular vesicles in which *TLR3, 7, 8*, and *9* are expressed include ER, endosomes, lysosomes, and endolysosomes. An excellent review of TLR expression and function is found in Ref. (17). The results presented in Figure 1C indicate the expression levels of TLRs in various tissue macrophages.

## NOD-Like Receptors or NBD–LRR Containing Proteins

This class of pathogen receptors/sensors (20) regulates a number of key inflammatory pathways. *NLRs* (Table 4) contain a nucleotide-binding domain (NBD) and leucine-rich repeats (LRR). These proteins are conserved in evolution from plants to humans. *NLR* proteins are subdivided by their N-terminus, which can contain acid transactivation, pyrin, CARD, or BIR domains. A major pathway is the activation of the inflammasome, which is a macromolecular structure that when assembled, causes the cleavage and activation of caspase-1, and thus can produce the active species of *IL1*β and *IL18*. There are 22 known human *NLRs*; here, only the best-studied will be discussed. Some, but not all *NLRs* assemble an inflammasome. Additionally, some inflammasome-inducing *NLRs* also exhibit inflammasome-independent functions. The molecular mechanisms of inflammasome activation have been reviewed elsewhere (20). This review will briefly describe the key *NLR* molecules important for macrophage sensing with a focus on the inflammasomes.

**Table 4:** **NOD-like receptors**.

Gene name	NLR	Pathogen or disturbance	Downstream effectors, function, and regulation
*Nlrp3*	NLRP3	Cellular stress	Activates caspase-1 through ASC
		ER stress	
		And ATP	Inhibited by NO from iNOS (S-nitrosylation)
		Monosodium urate, alum	
*Nlrc4*	NLRC4	Bacterial type III (T3SS) and IV (T4SS) secretion system, cytosolic flagellin	Activates caspase-1 to activate IL-1 and IL-18, blocks STING pathway
*Nlrc3*	NLRC3	HSV	Blocks STING pathway
*Nlrp1a*	NLRP1A	Lethal toxin (LT) of *Bacillus anthracis* (mouse)	
*Nlrp1b*	NLRP1B	Muramyl dipeptide (MDP) and titanium dioxide (human)	
*Nlrp1c*	NLRP1C		
*Nlrp6*	NLRP6 [PYPAF5]	Pathobiont bacteria, Bacteroidetes (Prevotellaceae), and TM7 microbiota	Blocks NFkB and MAPK pathway, activates caspase-1, inflammasome
*Nlrp7*	NLRP7	Mycoplasma acylated lipopeptide (acLP)	Inflammasome: hydatidiform
*Nlrp12*	NLRP12	*Yersinia*, malaria	Inflammasome function, inhibits NFkB and MAPK
*Nlrc5*	NLRC5	Bacteria, PAMPs, DAMPs, rhinovirus	Inflammasome function
			MHC class I gene expression
*Aim2*	AIM2	Cytosolic DNA	Activates Caspase-1 through ASC
*Pycard*	ASC	Common adaptor for inflammasome activation	Activates caspase-8 and -9
*Naip1*	NAIP1	NAIPs provide ligand specificity to the NLRC4 inflammasome.	Naip1, 2, 5, and 6 provide ligand specificity to the NLRC4 inflammasome. Specifically, Naip1 detects the bacterial rod, and Naip5 and 6 detect bacterial flagellins (61). The Naips are involved in expulsion of salmonella-infected enterocytes (62).
*Naip2*	NAIP2		
*Naip4*	NAIP4		
*Naip6*	NAIP6		

The best-studied inflammasome is anchored by *NLRP3*, a receptor that does not directly recognize DAMPs or PAMPs but rather responds to cellular stress, including changes in potassium efflux, ROS production, ER stress, and unfolded protein response. The adapter GBP5 promotes *NLRP3* inflammasome activation by ATP, nigericin, and bacteria, but not by crystalline agents. Several inhibitory pathways for *NLRP3* activation have also been described. For example, nitric oxide can cause the S-nitrosylation of *NLRP3* and impair assembly of the inflammasome (55–57). The G Protein signaling modulator-3 protein can associate with the LRR domain of *NLRP3* and inhibit inflammasome activation. cAMP binds and inhibits *NLRP3*, and the neurotransmitter dopamine negatively regulates NLRP3 function via cAMP that promotes *NLRP3* ubiquitination and subsequent degradation by the E3 ligase, *MARCH7* (58). *NLRP3* inflammasome activation depends on ASC (gene name *Pycard*), a common adaptor required for the activation of *caspase-1* by several inflammasomes, and is comprised of a pyrin domain and a CARD domain. Linear ubiquitination via the assembly complex LUBAC is known to enhance ASC function in the activation of *NLRP3*-dependent inflammasome (59). The *NLRP3* inflammasome also senses mitochondrial DNA. Activated *caspase-1* or the related *caspase-11* can induce pyroptosis (lytic cell death).

The *NLRC4* inflammasome activation (60) is enhanced by ASC, and it detects bacterial proteins in the cytosolic compartment, either as markers for the activity of bacterial type III and IV secretion systems or cytosolic invasion by flagellated bacteria. *NLRC4* responds to injection of three conserved bacterial proteins: flagellin, rod, and needle. NAIP proteins are also members of the NLR family. NAIP1, 2, 5, and 6 provide ligand specificity to the NLRC4 inflammasome. Specifically, NAIP1 detects the bacterial Needle protein, NAIP2 detects bacterial Rod, and NAIP5 and 6 detect bacterial flagellins (61). The NAIPs are involved in expulsion of *salmonella*-infected enterocytes (62).

*NLRP1 (DEFCAP, NAC, or NALP1)* is the *NLR* that defined the inflammasome (63). Mice express three highly polymorphic paralogs: *Nlrp1a, Nlrp1b*, and *Nlrp1c* due to likely gene duplication, while humans only express a single *NLRP1*. Mouse *Nlrp1b* and rat *Nlrp1*, but not human *NLRP1* mediates the response to the lethal toxin (LT) of *Bacillus anthracis*. Reminiscent of the requirement of a cleavage/activation process for *caspase-1*, *IL1*β, and *IL18* function, a maturation cleavage of the N-terminal of murine *NLRP1* is also required for its activation by LT (64). Among pathogens, *NLRP1* is also activated by *Toxoplasma gondii* and is required for host protection (65), although this process does not require the above-mentioned N-terminal cleavage (66). *NLRP1* is not the only *NLR* required for inflammasome activation by *T. gondii*, as *NLRP3* was also shown to play a role in host resistance to this pathogen (67).

*NLRP6* was originally found to synergize with ASC to cause *NFkB* and *caspase-1* activation in overexpression systems. *Nlrp6^−/−^* mice show spontaneous and induced colitis (68), associated with reduced *IL18* and expanded pathobiont bacteria, Bacteroidetes (Prevotellaceae), and TM7 microbiota (69). Other reports indicate alternative roles for the *NLRP6* in the control of intestinal cell apoptosis or as a checkpoint regulator of inflammatory mediators and the *NFkB/MAPK* pathways.

Other *NLRs* with inflammasome functions also have demonstrated *in vitro* relevance in other important pathways. For example, *NLRP7* mediates inflammasome activation by mycoplasma acylated lipopeptide in a human macrophage cell line, but it has been genetically linked in multiple patient cohorts (70) to the formation of hydatidiform moles, which is an abnormal form of pregnancy. *NLRP12* (*Monarch-1 or PYPAF7*) exhibits at least two functions as an inhibitor of the non-canonical and the canonical *NFkB* pathway. This was confirmed by *in vivo* studies of colitis-associated colon carcinoma models (71, 72), a bacterial infection model as well as a study of osteoclasts. *Yersinia* strains, but not several other bacteria, can cause *Nlrp12*-dependent inflammasome activation (73), and *Plasmodium* species induce a *NLRP12-* and *NLRP3*-dependent inflammasome activation (74). This dual requirement for two *NLR* is also observed with *NLRC5* and *NLRP3*, which interact with each other in a human macrophage cell line and PBMCs in response to a host of *NLRP3* activators (75). *Nlrc5^−/−^* mice show deficiencies in response to *NLRP3* activators, such as monosodium urate, alum, and ATP (76). However, the primary function of *NLRC5* is the regulation of class I MHC genes, thus demonstrating again the multi-faceted nature of *NLRs* (77).

In addition to the activation of caspase-1, a non-canonical pathway leading to *caspase-11* maturation that requires *NLRP3*, and can be activated by some bacteria, which appears to be detrimental during endotoxic shock (78). *Caspase-11* is activated by LPS from Gram-negative bacteria that invade the cytosol, and protects mice against lethal infection by *Burkholderia thailandensis* and *Burkholderia pseudomallei*. By contrast, vacuolar bacteria, such as *Salmonella typhimurium* are poorly detected by caspase-11. Caspase-11 is non-responsive, unless macrophages are primed by *IFN-*β or *IFN-*γ, both of which activate the transcription factor *STAT1*. *Caspase-11* responds to cytosolic lipid A species with five or six acyl groups, but not to species with four acyl groups. This expands the role of LPS as a key microbial pattern detected by the innate immune system: extracellular and vacuolar LPS is detected through *TLR4*, whereas cytosolic LPS is detected through *caspase-11*. Interestingly, both *Tlr4*- and *caspase-11*-deficient mice are resistant to classical LPS challenge (78). The results presented in Figure 1D reveal the expression levels of *NLR* and related molecules.

## Sensors for Intracellular RNA and DNA

RNA and DNA receptors recognize components of viral genomes. Herpes viruses such as herpes simplex or murine cytomegalovirus (MCMV) are double-stranded (ds)DNA viruses, where the dsDNA is recognized by *STING* or *IFI16* (mouse gene name *Ifi204*). Single-stranded (ss) viral RNA often forms stem–loop structures that can be detected by *TLR7* (see Toll-Like Receptors) and *RIG-I* (79). ssDNA (not necessarily viral) is recognized by *TLR9*, especially when it is CpG-rich and not methylated.

These receptors are expressed in macrophages, epithelial cells, and other cells. The immune response starts in infected cells, where the viruses replicate in the cytoplasm. The intracellular viral RNA is detected by the *RIG-I* family of receptors, including *RIG-I* (gene name: *Ddx58*), *MDA5* (*Ifih1*), and *LGP2* (*Dhx58*). *RIG-I* detects many ssRNA viruses, including influenza virus, hepatitis C, Dengue, yellow fever, west Nile, and Ebola. The *RIG-I* family receptors require *MAVS* as an adapter protein and *IRF3* or *IRF7* as downstream transcription factors, resulting in the elaboration of *IFN-*α and β.

Single-stranded DNA is recognized by *TLR9* (see Toll-Like Receptors). DNA-derived intracellular cyclic dinucleotides are recognized by *STING* (gene name, *Tmem173*) (80). Humans have five common *STING* alleles. Listeria products are processed by cGAS (cyclic GMP AMP synthetase), which produces cGMP that in turn activates *STING*. *STING* is also involved in detecting herpes virus. Other sensors for intracellular DNA are *DAI* (gene name, *Zbp1*), *Aim2* (see under *NLRs*), *DDX41* (*Ddx41*), *DHX9* (*Dhx9*), *IFI16* (gene name, *Ifi204*), and *DHX36* (*Dhx36*). *DAI* senses dsDNA. The results in Figure 1E show the mRNA expression of these DNA- and RNA-sensing receptors in various tissue-resident macrophages. *Aim2* expression was only detected in bone marrow-derived macrophage (BMDM), peritoneal macrophages, and microglia.

## C-Type Lectin Receptors

In general, C-type lectin receptors (Table 5) recognize multivalent carbohydrate ligands (4) that might be present on endothelial cells, epithelial cells, pathogens, and other microorganisms. C-type lectins are also involved in the uptake and clearance of dead cells (6). Macrophages express several C-type lectin receptors, which include collectins, selectins, lymphocyte lectins, and proteoglycans (4). Some are important sensors for fungal infections (81), others such as the selectins recognize host glycans (82). The C-type lectins *DCIR* and *MICL* are inhibitory, because their cytoplasmic tails contain ITIM domains. *Dectin-2*, *Mincle*, and *DCAR* are coupled to an ITAM-domain containing adaptor. *Dectin-2* is the prototypic receptor of the *Dectin-2* subfamily of C-type lectins that also contains *CLECSF8*, *DCIR* and *Mincle* and *DCIR2*, *3*, and *4*, *DCAR*, and *DCAR1* in mice only (5). *SignR3*, *Dectin-1*, and *CLEC9a* also contain at least one ITAM domain. *DC-Sign, SIGN-R1, Langerin, MGL, DEC-205*, and the mannose receptor MR are not coupled to either ITAM or ITIM domains, but signal through adaptor proteins.

**Table 5:** **C-type lectins**.

Gene name	C-type lectin	CD	Binds ligands from	Function
*Ly75*	DEC-205	CD205	HIV, *Yersinia pestis*, *E. coli*; endogenous	Uptake of pathogens and apoptotic cells
*Clec10a*	Mgl1	CD301a	Streptococci, Lactobacilli, apoptotic cells: Sialoadhesin	Uptake of pathogens and apoptotic cells
*Clec9a*	DNGR-1		F-actin	Uptake of necrotic cells
*Clec4e*	Mincle		α-mannose and glycolipids from *Mycobacterium tuberculosis*, *Candida albicans*	Necrotic cell uptake; couples through FcRγ, activates NFkB
*Clec7a*	BGR	CD369	Beta-1,3-linked and beta-1,6-linked glucans from fungi and plants	
*Clec4a1*	DCIR4		Unknown	
*Clec12a*	MICL	CD371		Down regulated by cell activation
*Clec4n*	Dectin-2-like			
*Clec4b1*	DCAR			Associates with Fc receptor γ chain
*Clec4d*	MCL, MPCL	CD368		Endocytotic, signals through Syk
*Clec4a3*	DCIR3			
*Clec4a4*	DCIR2		Bisecting *N*-acetylglucosamine	
*Clec4k*	Langerin	CD207	Measles virus	
*Cd209a*	DC-SIGN	CD209	Microbial polysaccharides, sialylated antibody	Possible role in Th17 response
*Cd209b*	SIGNR1, CLEC4m	CD209b	Oligomannose	Interacts with C1q
*Cd209d*	SIGNR3	CD209d	*Leishmania*, mycobacterial saccharides	
*Clec1b*	Clec2		High on platelets	Required for lymphangiogenesis, separates lymphatics from blood vessels
*Sell*	L-selectin	CD62L	High endothelial venules, PSGL-1	Rolling of Ly6C^hi^ monocytes
*Selp*	P-selectin	CD62P	PSGL-1, TIM1	Endothelial cells: rolling; macrophages: unknown
*Sele*	E-selectin	CD62E	PSGL-1, CD44, CD43, others	Largely restricted to endothelium

E-selectin recognizes fucosylated and sialylated ligands of the sialyl-Lewis^x^ type. P-selectin recognizes the glycoproteins PSGL-1 on myeloid cells and TIM1 on activated T cells. L-selectin recognizes 6-sulfated carbohydrates expressed in high endothelial venules and PSGL-1. Many macrophages express P-selectin, but its function in macrophages has not been studied. Monocytes express L-selectin, but its expression is lost upon differentiation to macrophages. L-selectin is an excellent example for an adhesion molecule that is shed upon cell activation (83). Shedding of adhesion molecules, cytokine receptors, and pattern recognition receptors is an important mechanism that limits their signaling (84, 85). In some cases, the shed molecules can have signaling functions in their soluble form. The results in Figure 1F demonstrate the expression levels of C-type lectins.

## Scavenger Receptors

Scavenger receptors (Table 6) are cell surface receptors that typically bind multiple ligands and promote the removal of non-self or altered self targets. Both tissue-resident and monocyte-derived macrophages express a number of scavenger receptors. They often function by mechanisms that include endocytosis, phagocytosis, adhesion, and signaling that ultimately lead to the elimination of degraded or harmful substances (7). Scavenger receptors recognize non-self (mostly bacterial products) and altered (oxidized, acetylated) self. They are more involved in uptake and phagocytosis than activation of inflammatory effector systems. Scavenger receptors have recently been classified (53). The systematic names are SR followed by a letter (for the class) and numbers (for the members).

**Table 6:** **Scavenger receptors**.

Gene name	Systematic name	Other names	Ligands and function
*Msr1*	SR-A1	SCARA1 MSR1	Heat shock proteins, acLDL, oxLDL Bacteria, yeast, amyloid, hepatitis C virus
*Scara3*	SR-A3	SCARA3 CSR	Protection from ROS
*Colec12*	SR-A4	SCARA4 SRCL, COLEC12	Modified LDL Endocytic receptor for lipoproteins
*Scara5*	SR-A5	SCARA5	Binds bacteria
*Marco*	SR-A6	MARCO	Bacterial clearance from lung and blood
*Scarb1*	SR-B1	SCARB1	HDL
*Cd36*	SR-B2	CD36 Scarb3	Thrombospondin [modulates angiogenesis], foam cell formation in atherosclerosis by uptake of oxLDL, binds fungi, bacteria, *Plasmodium falciparum*-infected RBC interacts with TLR4 and TLR6, uptake of apoptotic cells
*Scarb2*	SR-B3	LIMP2	Delivers β-glucocerebrosidase from the endoplasmic reticulum to lysosomes, enterovirus 71 (EV71), coxsackievirus 7 (CVA7), CVA14, CVA16
*Cd68*	SR-D1	Macrosialin CD68	Controversial: may bind oxidized lipoproteins, apoptotic cells, but inconsistent data
*Olr1*	SR-E1	LOX-1 OLR1	oxLDL, CRP
*Clec7a*	SR-E2	Dectin-1	Bacterial, fungal, and plant carbohydrates β-glucans interacts with TLR2
*Scarf1*	SR-F1	SCARF1 SREC-I	Calreticulin, fungal and heat shock proteins, cross-presentation of antigens on MHC-I. Binds oxLDL. Apoptotic cell clearance
*Scarf2*	SR-F2	SREC-II	
*Megf10*	SR-F3	MEGF10	Amyloid-β
*Cxcl16*	SR-G1	RS-PSOX CXCL16	oxLDL, phagocytosis of bacteria, chemotaxis through CXCR6
*Stab1*	SR-H1	Stabilin-1 FEEL-1 CLEVER-1	Lymphocyte adhesion, transmigration, angiogenesis, apoptotic cell clearance, and intracellular trafficking
*Stab2*	SR-H2	Stabilin-2 FEEL-2	Binds PS, interacts with GULP and thymosin-β4 to initiate uptake of apoptotic cells
*Cd163*	SR-I1	CD163	Hemoglobin-haptoglobin, anti-inflammatory signaling, secretion of cytokines IL-6 and IL-10
*Ager*	SR-J1	RAGE	Advanced glycation end products, high mobility group protein box 1, S-100, oxidative stress, pro-inflammatory
*Lgals3bp*	Galectin-3 binding protein	GAL3BP	Binds galectin-1 and 3, anti-inflammatory, produced by M1 macrophages

*SR-A1*, *2*, and *3* and *MARCO* form the SR-A subfamily. Only *SR-A1* has an SRCR domain and is the main macrophage receptor for HDL. *MARCO* is a collagen-like molecule; *MARCO* defects result in susceptibility to tuberculosis. The *SR-B* subfamily comprises *SR-B1* and *SR-B2* (*CD36*). *CD36* is an important receptor for diacyl glycerides, oxidized LDL, and the malaria parasites. *CD36* is important for ROS and cytokine production by macrophages. There are no mammalian members of the *SR-C* subfamily. *SR-D* is *CD68* (macrosialin), an intracellular receptor of unknown function. *SR-E1* is *Lox1* (Loxin), which detects oxidized LDL. *Dectin-1* is *SR-E2*. The mannose receptor MR is *SR-E3* and the asialoglycoprotein receptor is *SR-E4*. *SR-F1* is *SCARF1*, *Ced1* in *Caenorhabditis elegans*, and *SR-F2* is *SCARF2*. The only known *SR-G* is *CXCL16*, a receptor for oxidized LDL and a chemokine that binds *CXCR6*. *SR-H1* is *FEEL-1*, also known as *Stabilin-1* or *Clever-1*, *SR-I* is *CD163*, the macrophage receptor for hemoglobin–haptoglobin complexes. In bovine and ovine species, this subfamily has many more members. *SR-J* is *RAGE*, a seven-transmembrane receptor with multiple immunoglobulin domains that sees glycated proteins and advanced glycation end products. *SR-L1* is also known as *LRP1*, and *SR-L2* as *LRP-2*.

Some macrophage scavenger receptors are currently unclassified. Galectin-3-binding protein is a secreted product of M1 macrophages that binds galectin-3 and other cell surface ligands. *CD207* (Langerin), *MRC1* (*CD206*), *CLEC7A*, and *CD209* (DC-SIGN) are also unclassified. *CD206* is prominently expressed on M2 macrophages. C1q interacts with the Aβ protein relevant in Alzheimer’s disease. The results in Figure 1G show the expression levels of scavenger receptors.

## Cytokine Receptors

Another class of macrophage receptors sense products of adaptive immune cells. These receptors can be considered secondary amplifiers, because they do not detect non-self or altered self, but rather amplify the macrophage response. Since most cytokines are produced by activated T cells that require antigen presentation and an ongoing immune response, the cytokine receptors on macrophages sense the nature of that ongoing immune response (Th1, Th2, Th17) (Table 7).

**Table 7:** **Cytokine receptors on macrophages**.

Gene name	Common name	CD	Ligands and function
*Ifngr1*	IFN-γ R		IFN-γ: forces M1 polarization
*Il1r1*	IL-1 R1	CD121a	IL-1α and β, pro-inflammatory
*Il1r2*	IL-1 R2	CD121b	IL-1α and β, pro-inflammatory
*Il4ra*	IL-4 R	CD124	IL-4: forces M2 polarization
*Il5ra*	IL-5 R	CD125	IL-5: forces M2 polarization
*Il10ra* *Il10rb*	IL-10Rα IL-10Rβ	CD210	IL-10: strongly anti-inflammatory, suppresses antigen presentation (135)
*Il13a1*	IL-13 R	CD213a	IL-13: forces M2 polarization
*Il13a2*	IL-13 R	CD213b	IL-13: forces M2 polarization
*Il17ra*	IL-17RA	CD217	IL-17a: activates macrophages
*Il17rb*	IL-17RB		Basophil function, asthma
*Il17rc*	IL-17RC		Needed for IL-17A-induced CXC chemokine expression
*Il17rd*	IL-17RD		Anti-inflammatory
*Il17re*	IL-17RE		Receptor for IL-17C: immunity to intestinal pathogens
*Il27ra*	IL-27R		IL-27: regulates Th1 and Treg
*Tgfbr1*	TGF-βR1		TGF-β: inactivates macrophages, determining receptor of microglia
*Tgfbr2*	TGF-βR2		TGF-β: inactivates macrophages
*Tgfbr3*	TGF-βR3		TGF-β: inactivates macrophages

IFN-γ receptor is a major activator of macrophages and reinforces the M1 phenotype (12). The *Ifngr1* gene encoding this receptor is highly expressed in all macrophages. Its functional antagonist is IL4 receptor. Because of their important role in inflammation, we also discuss IL1 receptors, IL17 receptors, and IL27 receptor. A major anti-inflammatory pathway is initiated by TGF-β, which has three receptors.

IL4, 5, and 13 receptors promote and reinforce the M2 phenotype (3, 86, 87), but their ligation is not required for M2 polarization; in fact, it appears that M2 is the default “healing” phenotype of macrophages (5). IL13 receptor α1 chain is expressed in all macrophages, but the α2 chain is missing from peritoneal macrophages. IL5 receptor is expressed at very low levels on these cells.

TGF-β receptors strongly promote M2 polarization. *TGF-*β is made by many tissue cells, especially epithelial cells, and seems to signal to the macrophage that the tissue cells are in good health and not infected. *Tgfbr1* is highly expressed in all macrophages, with highest levels in microglia. The same is true for *Tgfbr2*, but with highest levels in adipose tissue macrophages. *Tgfbr3* is expressed in all macrophages except microglia and BMDM. Overabundance of *TGF-*β can lead to excessive collagen formation resulting in fibrosis. Macrophages have receptors for cytokines of the IL17 family. The results in Figure 1L indicate that the *IL17* receptor A is expressed in all macrophages, and being highest in microglia. *Il17rb*, *c*, *d*, and *e* are expressed at low levels. *IL1* receptors 1 and 2 are expressed at high levels in all macrophages.

## TNF Receptor Superfamily

The TNF receptor superfamily (*Tnfrsf*) (88–91) bind soluble and cell surface-expressed TNF superfamily members that have diverse biological functions (Table 8). These can range from molecules that induce caspase activation and apoptosis and regulate cell death (Fas, TNFRI, TRAILR) to those that activate NFκB and MAPK pathways and are pro-inflammatory (e.g., TNFRII, RANK, CD40, BAFFR, OX40) inducing a multitude of effects, such as promoting division, survival, cytokine production, chemokine production, and upregulation of other receptors in varied protein families. The summarized expression values in Figure 1I reveal that the Tnfrsf receptors are of specific interest here, because many of them show highly differential expression among tissue macrophages, suggesting that different macrophage subsets have different abilities to see Tnfsf ligands.

**Table 8:** **TNF receptor superfamily**.

Gene name	Systematic name	Other names	Ligands and function
*Tnfrsf1a*	Tnfrsf1a	TNFR1	TNF, soluble lymphotoxin α: pro- and anti-inflammatory (apoptosis) many cell types
*Tnfrsf1b*	Tnfrsf1b	TNFR2	TNF, soluble lymphotoxin α: pro-inflammatory many cells; T cell activation
*Ltbr*	Tnfrsf3	LTβR	Membrane lymphotoxin β and LIGHT: pro-inflammatory for APC and tissue cells; lymph node development
*Tnfrsf4*	Tnfrsf4	OX40, CD134	OX40L (=CD252): T and NK activation/differentiation
*Cd40*	Tnfrsf5	CD40	CD40L (=CD154): immunoglobulin class switching, APC activation
*Fas*	Tnfrsf6	Fas CD95	FasL (=CD178): apoptosis/cell death
*Cd27*	Tnfrsf7	CD27	CD70: T and B cell activation; antibody production
*Tnfrsf8*	Tnfrsf8	CD30	CD30L (=CD153): T and B cell activation
*Tnfrsf9*	Tnfrsf9	4-1BB, CD137	4-1BBL: T and NK activation/differentiation
*Tnfrsf10b*	Tnfrsf10b	TRAIL R2 DR5 CD262	TRAIL (=CD253): apoptosis in tumor cells and T cells
*Tnfrsf11a*	Tnfrsf11a	RANK CD265	RANKL (=CD254): osteoclast and lymph node development; activation of APC. Blockers of RANKL (antibody: denosumab) binding to RANK are used clinically to treat osteoporosis
*Tnfrsf11b*	Tnfrsf11b	OPG	Decoy receptor for RANK: modulates osteoclastogenesis
*Tnfrsf12a*	Tnfrsf12a	Fn14 CD266	TWEAK: tissue inflammation
*Tnfrsf13b*	Tnfrsf13b	TACI, CD267	APRIL (= CD256) and BAFF (= CD257): B cell activation/differentiation
*Tnfrsf13c*	Tnfrsf13c	BAFFR CD268	BAFF (= CD257): B cell differentiation/survival. Blockers of BAFF (antibody: belimumab) binding to its receptors BAFFR, TACI, and BCMA are used clinically to treat systemic lupus.
*Tnfrsf14*	Tnfrsf14	HVEM CD270	LIGHT (= CD258): T cell activation; pro-inflammatory tissue cells and APC; BTLA: T cell inhibition
*Tnfrsf17*	Tnfrsf17	BCMA CD269	APRIL (= CD256) and BAFF (= CD257): B cell activation/differentiation
*Tnfrsf18*	Tnfrsf18	GITR CD357	GITRL: T and B cell activation
*Tnfrsf19*	Tnfrsf19	TROY	Nogo coreceptor: axon regeneration? Unknown: hair follicle development?
*Tnfrsf21*	Tnfrsf21	DR6 CD358	Amyloid precursor protein: neuron death? Unknown: T cell inhibition/death
*Tnfrsf22* *Tnfrsf23*	Tnfrsf22 and 23	mDcTRAIL R2 and mDcTRAILR1	Decoy receptors for TRAIL only expressed in mice: neutralize TRAIL activity
*Tnfrsf25*	Tnfrsf25	DR3	TL1A: T cell activation/differentiation
*Eda2r*	Tnfrsf27	XEDAR EDA2R	EDA: control of hair follicle, sweat gland, teeth development

Many of the *Tnfrsf* members are well known for controlling responses of T and B cells, dendritic cells, NK, and NKT cells, as well as inflammatory activity in structural cells, such as fibroblasts, epithelial cells, and osteoclasts. This has resulted in clinical targeting of many of the molecules for autoimmune and inflammatory diseases (89), and approved drugs in blockers of TNF and LTα binding to TNFRI and TNFRII for RA, psoriasis, Crohn’s disease, and others; blockers of RANKL binding to RANK for osteoporosis; and blockers of BAFF binding to its receptors BAFFR, TACI, and BCMA for SLE.

In macrophages, there appears to be strongly divergent expression among tissue macrophages (Figure 1I). *Tnfrsf1a* (TNFR1), *Tnfrsf1b* (TNFR2), *Tnfrsf11a* (RANK), and *Tnfrsf3* (LTβR) are highly expressed by most tissue macrophages. *Tnfrsf5* (CD40), *Tnfrsf12a* (Fn14), *Tnfrsf14* (HVEM), *Tnfrsf21* (DR6) are also highly expressed in some macrophage populations, but variably expressed in others at moderate levels. TNFR2 is largely pro-inflammatory, whereas TNFR1 can display both pro-inflammatory and anti-inflammatory (apoptosis-inducing) activities. Since TNF is a product of macrophages, various reports have suggested both positive and negative effects of TNF on macrophages correlating with these functions of its receptors (92–96). The outcome of TNFR signaling in macrophages may be heavily influenced by the location of the cells and the inflammatory environment in which they are responding. CD40, RANK, and LTβR are stimulatory receptors for antigen-presenting cells, particularly dendritic cells and B cells. CD40 is best known in this regard, but RANK and LTβR can display overlapping and synergistic activities. They have been less studied in macrophages, but parallel activities in promoting survival, differentiation, and inflammatory cytokine production, as well as upregulating co-stimulatory ligands that promote T cell activation, are consequences of engaging these receptors (97, 98). However, some studies of LTβR signaling on macrophages have suggested a regulatory activity in limiting TLR signaling (99). HVEM shares binding to the ligand LIGHT with LTβR. Signaling from either HVEM or LTβR can promote migration of macrophages (98, 100) and can induce inflammatory mediators, such as TNF, IL8, MMPs, and TGF-β (101, 102). Fn14 has been implicated in driving inflammation and remodeling activities in several tissues. TWEAK, the ligand for Fn14, can be made by macrophages (103) and TWEAK can also exert pro-inflammatory activity in macrophages *via* Fn14, promoting production of molecules, such as IL6, IL8, MCP-1, MMPs, and HMGB1, and driving macrophage migration into inflamed sites (104–107).

The receptors for BAFF and APRIL are best known as regulators of B cell survival or differentiation and also control T cell activity in some cases. *Tnfrsf13c* (BAFFR) that only binds BAFF was low in all macrophages, but *Tnfrsf13b* (TACI) and *Tnfrsf17* (BCMA) that bind BAFF and APRIL were high in intestinal, adipose, and peritoneal macrophages and adipose macrophages and microglia, respectively. BAFF can be made by macrophages in soluble and membrane form, and membrane BAFF may (reverse) signal to promote inflammatory mediators (108), but the roles of the receptors are not well understood. Recent studies suggest that TACI or BCMA signals can also promote pro-inflammatory cytokines/mediators (109). TACI activity may also suppress differentiation of macrophages into the M2 phenotype, allowing enhanced clearance of intracellular organisms (110).

*Tnfrsf9* (4-1BB), *Tnfrsf25* (DR3), *Tnfrsf4* (OX40), *Tnfrsf18* (GITR), *Tnfrsf8* (CD30), and *Tnfrsf7* (CD27) showed low levels of transcript expression in most tissue macrophages, as did the decoy receptor for RANK (*Tnfrsf11b* – OPG). 4-1BB, DR3, OX40, GITR, CD30, and CD27 are best known as regulators of conventional T cells as well as Treg cells, NK, or NKT cells. Most have rarely been found on the surface of macrophages, although several studies have seen DR3 and GITR on macrophages/foam cells associated with atherosclerotic plaques, or in joints associated with RA (111–114). Signaling through these receptors, probably via NFkB, may aid production of pro-inflammatory molecules, such as TNF or MMPs. The ligands for 4-1BB, DR3, OX40, GITR, and CD27 can be expressed by many macrophages after they are activated, and can aid the antigen-presenting capacity of macrophages by providing co-stimulatory signals to their receptors on T cells. Certain TNF family ligands, like 4-1BBL, TL1A (ligand for DR3), and GITRL can also signal into macrophages or monocytes (reverse signaling) to modulate their survival, production of cytokines or molecules, such as PGE2, COX-2, and iNOS, and in some cases migration (115–119). In the case of 4-1BBL, it can also associate with TLRs on macrophages independently of binding its receptor and enhance TLR signaling (120, 121).

*Tnfrsf27* (EDA2R) was low/absent in most macrophages, not surprising given its role outside the immune system in formation of tissues, such as sweat glands. *Tnfrsf19* (TROY) is primarily expressed in the nervous system and has been suggested to regulate axon regeneration, but may also contribute to hair follicle formation. TROY was also low in most tissue macrophages and BMDMs. One prior report had suggested that it could be expressed in microglia in MS patients (122) but this has not been confirmed.

The death receptors *Tnfrsf10b* (TRAILR2) and *Tnfrsf6* (Fas), and the mouse decoy receptors for TRAIL (*Tnfrsf22* and *23* – mDcTRAILR1 and R2) showed low levels of expression in most macrophages, except for Fas in peritoneal macrophages and mDcTRAILR1 in BMDM, lung, liver, and peritoneal cells. Fas is likely to promote apoptosis of macrophages in some cases to limit autoimmunity or inflammation (123, 124), but it can be pro-inflammatory by augmenting caspase-dependent production of *IL1* and *IL18* or other activities (125–127). It is not known when production of the TRAIL decoy receptor by macrophages could be important for limiting TRAILR activity. *Tnfrsf21* (DR6) is another death receptor that has been reported to regulate neuronal death and may also limit T and B cell activation, but whether it has other functions is not clear. It was highly expressed in microglia, peritoneal, spleen, and lung macrophages and BMDM (Figure 1I).

## Fc Receptors

Fc receptors (Table 9) are receptors for immunoglobulins, which are products of plasma cells, and immune complexes. There are receptors for IgG (*Fc*γ*RI, II*, and *III*) (128, 129) and IgE (*Fce receptor*). They vary in their affinity (high to low) and in their downstream signaling effects (activating and inhibitory). Fc receptors are very highly expressed and are of key importance for clearing antibody-opsonized pathogens and antibody-opsonized necrotic cells. In the mouse, most Fc receptors signal through a common adaptor protein called Fc receptor γ chain or CD23.

**Table 9:** **Fc receptors**.

Gene name	FcR	CD	Function
Fcgr1	FcRγI	CD64	High affinity IgG binding
Fcgr2b	FcRγIIb	CD32	Low affinity IgG R, immune complexes
Fcgr3	FcRγIII	CD16	Low affinity IgG R, immune complexes
Fcgr4	FcRγIII-2	CD16-2	Low affinity IgG R, immune complexes
Fcer1a	FceR		High affinity IgE receptor
Fcer1g	Fc γ chain	CD23	Adaptor for most Fc receptors
Fcer2a	FcreR2	CD23a	Low affinity IgE receptor
Ms4a2	Fcer1b		

The results in Figure 1J summarize the expression of Fc receptors in tissue macrophages. *Fcgr1*, the gene encoding the CD64 Fc γ receptor, is specific for macrophages and has been proposed as a universal mouse macrophage marker (40).

## Chemokine Receptors and Other GPCRs

Macrophages and dendritic cells are important sources of many inflammatory chemokines including *CCL1*, *2*, *3*, *4*, and *5*. However, they also express chemokine receptors (Table 10) and, thus, respond to their chemokine environment. The major chemokine receptors on macrophages are *CCR2*, *CCR5*, and *CX3CR1* (130, 131). *CCR2* binds *CCL2* and related chemokines of the MCP subfamily and is responsible for release of monocytes from the bone marrow and their trafficking to inflamed tissues. *CCR1* and *2* are expressed in all tissue macrophages, but very low in microglia. *CCR5* is a receptor for *CCL5* and attracts monocytes to the vascular wall. It is expressed in all macrophages, highest in microglia. *CX3CR1* is a receptor for *CX3CL1* (fractalkine) and promotes macrophage survival. It is expressed in all macrophages, highest in microglia and intestinal macrophages.

**Table 10:** **GPCRs expressed on macrophages**.

Gene name	GPCR	CD, other	Function
*Ccr1*	CCR1	CD191	Pro-inflammatory
*Ccr2*	CCR2	CD192	Recruits monocytes
*Ccr5*	CCR5	CD195	Arrest, integrin activation, recruits monocytes
*Ccr7*	CCR7	CD197	Directs macrophages and dendritic cells to lymphatics and lymph nodes, also involved in lymphocyte homing and development
*Cx3cr1*	CX3CR1		Fractalkine R: Macrophage survival
*Adora1*	Adenosine A1		Pro-inflammatory, sleep-inducing
*Adora2a*	Adenosine A2a		Anti-inflammatory. Istradefylline is an A2A antagonist approved for Parkinson’s disease
*Adora2b*	Adenosine A2b		Pro-inflammatory
*Adora3*	Adenosine A3		
*P2ry1*	ATP R 1		ATP and ADP receptor
*P2ry2*	ATP R 2		Apoptotic cell recognition
*P2ry12*	ATP R 12		ATP and ADP receptor
*S1pr1*	S1P1	CD363	Regulates lymphocyte trafficking
*S1pr2*	S1P2	EDG5	
*S1pr3*	S1P3	EDG3	
*S1pr4*	S1P4	EDG6	

## Other GPCRs

Other GPCRs (Table 10) include four adenosine receptors, *Adora 1*, *2a*, *2b*, and *3*. Adenosine is a ubiquitous metabolite found in the extracellular space, and macrophages can respond to adenosine through these receptors. ATP is released by apoptotic cells through a pannexin-dependent mechanism, and ATP and ADP are important metabolites influencing macrophages through P2Y receptors. Sphingosine-1-phosphate is produced by platelets and other blood cells and is sensed by macrophages through *S1P* receptors *S1P1-4* (Figure 1K).

The adenosine receptor A2A (*Adora2a*) is expressed at low levels in all tissue macrophages except microglia. This receptor downregulates inflammatory responses. A2B (*Adora2b*) is also expressed at low levels, but much higher in adipose tissue macrophages. This is considered a pro-inflammatory receptor.

*P2Y1* (*P2ry1*) is an ADP receptor with low expression in all macrophages except microglia, where it does not appear to be expressed. By contrast, the ADP receptor *P2Y12* (*P2ry12*) shows low expression in all macrophages but very high expression in microglia. *P2Y12* on platelets is a target for anti-thrombotic treatment. *P2Y2* is a receptor for UTP and ATP, which is released from apoptotic cells via pannexin (*PANX1*) (132).

The sphigosine-1-phosphate receptor encoded by *S1pr1* is expressed by all tissue macrophages. *S1pr2* is also broadly expressed, with highest levels in BMDM and bone marrow macrophages. *S1pr3* and *S1pr4* are expressed at low levels. Expression of these GPCRs is shown in Figure 1K.

## Integrins

Macrophages use integrins (Table 11), αβ heterodimers that bind to extracellular matrix molecules (ECM) and other cells (133, 134). Since integrins, once bound to ligand, promote outside-in signaling, integrin engagement is an important input into macrophage biology. Almost all integrin subunits are expressed at some level in various tissue macrophages.

**Table 11:** **Integrins**.

Gene Name	Integrin	CD, other	Function
*Itgb1*	β1 integrins	CD29	Pairs with α1–11
*Itga1*	α1β1	CD49a	Collagen receptor
*Itga3*	α3β1	CD49c	Type 4 collagen receptor
*Itga4*	α4β1	CD49d	Binds VCAM-1 and alternatively spliced fibronectin
		VLA-4	
*Itga5*	α5β1	CD49e	Bind fibronectin, pro-inflammatory signal
		VLA-5	
*Itga6*	α6β1	CD49f	Laminin binding
		VLA-6	
*Itga7*	α7β1		Muscle development
*Itga8*	α8β1		Extracellular matrix assembly?
*Itga9*	α9β1		Fertilization of egg
*Itga10*	α10β1		Collagen binding
*Itga11*	α11β1		Collagen binding
*Itgb2*	β2 integrins	CD18	Pairs with αL, αM, αX, αd
*Itgal*	αLβ2	CD11a/CD18 LFA-1	Adhesion to ICAM-1, 2, and 3
*Itgam*	αMβ2	CD11b/CD18 Mac-1	Binds C3bi, ICAM-1, denatured proteins, and many other ligands
*Itgax*	αXβ2	CD11c/CD18	Binds C3bi, ICAM-1, denatured proteins
*Itgad* *Itgb3*	αDβ2 β3 integrins	CD11d/CD18 CD61	Binds ICAM-3
*Itga2b*	αIIbβ3	CD41/CD61	High on platelets, binds fibrinogen
*Itgav*	αVβ3	CD51/CD61	Major receptor for vitronectin, uptake of apoptotic cells
*Itgb6*	αVβ6	CD51	Involved in TGFβ activation (136)
*Itgb7*	β7 integrins	Ly69	Intestinal specific
*Itga4*	α4β7	CD49d LPAM	Binds MAdCAM-1
*Itgae*	αEβ7	CD103	Binds to E-cadherin

Many integrin α subunits pair with β1, which is expressed in all macrophages (*Itgb1*). *Itga1, Itga11, Itga2, Itga2b, Itga3*, and *Itga7* are expressed at low levels. *Itga4* pairs with *Itgb1* forming VLA-4, an integrin known to be involved in monocyte recruitment to tissues. *Itga4* expression varies widely between tissue macrophages, with highest expression levels seen in large and small peritoneal macrophages. *Itga5* pairs with Itgb1 to form a fibronectin receptor that is expressed at moderate levels in all tissue macrophages, highest in thioglycollate-elicited peritoneal macrophages. *Itga6/Itgb1* encodes a laminin receptor that is highly expressed in peritoneal macrophages and microglia, but lower in liver, spleen, and adipose tissue macrophages. *Itga8* is expressed in all tissue macrophages except peritoneal. *Itga9* and *10* are expressed at intermediate to high levels in all macrophages.

The integrin subunits *Itgal*, *Itgam*, *Itgax*, and *Itgad* all pair with *Itgb2* and are leukocyte specific. These β2 integrins are highly expressed in all macrophages, with highest mRNA levels in thioglycollate-elicited peritoneal macrophages. *Itgal* (LFA-1) is expressed at modest levels in most macrophages but not in microglia. *Itgam* (Mac-1), also known as complement receptor-3, is expressed at intermediate levels in most macrophages but very high in peritoneal and BMDM. *Itgax* (CD11c) is widely used as a dendritic cell marker, but this integrin is also expressed in all macrophages, most highly in thioglycollate-elicited peritoneal macrophages. *Itgad* (CD11d) is expressed at very low levels except for spleen macrophages, where it is very high.

Itgav pairs with *Itgb3* to form the vitronectin receptor α*V*β*3*, also known as leukocyte response integrin. Both subunits are expressed across all macrophages. *Itgav* can also pair with *Itgb6* to form α*V*β*6* integrin, which is involved in *TGF-*β processing and fibrosis. *Itgae* and *Itga4* pair with *Itgb7*, a receptor family generally found more in intestinal tissues. Indeed, *Itgb7* expression is highest in peritoneal and intestinal macrophages. *Itgae* is highly specialized and binds to E-cadherin. Its expression is low except in intestinal macrophages, where it is very high (Figure 1H). The collagen receptor α*2*β*1* integrin was not expressed in any of the macrophages studied here.

## Concluding Remarks

This review provides a broad overview of receptors used by macrophages to sense their environment, including apoptotic and necrotic cells, pathogens, carbohydrates, modified self molecules, cytokines, chemokines, and other soluble molecules and extracellular matrix components. Some of these macrophage receptors are broadly expressed in all tissue macrophages studied, while others are highly restricted. This suggests that certain macrophages are well equipped for certain functions (listed in the tables) and others are “blind” to certain challenges or inputs. Recent reviews on each of the 12 classes of receptors are cited in the respective subsections for more detailed information. The remarkable heterogeneity in expression of macrophage receptors suggests highly specialized functions of resting tissue macrophages in lung, liver, spleen, intestine, fat tissue, the peritoneal cavity, and the brain (microglia).

This review is limited to mRNA expression data, and it is unknown how these expression patterns correlate with protein levels. In macrophages, the correlation between mRNA and protein levels is modest. Also, we consider the 12 receptor classes individually, but they may cooperate or even compete for ligand recognition, which would affect macrophage function. There could be more than one “danger” that affects a tissue, such as infection with multiple pathogens or viral infection and apoptotic cells. How tissue macrophages respond to such complex inputs remains to be studied. We did not analyze infiltrating monocyte-derived macrophages here, which can be a dominant population in inflammatory diseases, infections, autoimmune diseases, and cancer.

## Author Contributions

KL wrote the paper. AP constructed the heat maps and input to the tables. MC wrote and edited the TNFSF part. KR edited the apoptotic cell uptake part. JT wrote and edited the NLR and RLR part.

## Conflict of Interest Statement

The authors declare that the research was conducted in the absence of any commercial or financial relationships that could be construed as a potential conflict of interest.

## Footnotes

^1^http://www.ncbi.nlm.nih.gov/geo/

^2^http://www.r-project.org
